# The Spatiotemporal Role of COX-2 in Osteogenic and Chondrogenic Differentiation of Periosteum-Derived Mesenchymal Progenitors in Fracture Repair

**DOI:** 10.1371/journal.pone.0100079

**Published:** 2014-07-02

**Authors:** Chunlan Huang, Ming Xue, Hongli Chen, Jing Jiao, Harvey R. Herschman, Regis J. O'Keefe, Xinping Zhang

**Affiliations:** 1 Center for Musculoskeletal Research, University of Rochester, School of Medicine and Dentistry, Rochester, New York, United States of America; 2 Department of Molecular and Medical Pharmacology, David Geffen School of Medicine, University of California Los Angeles, Los Angeles, California, United States of America; Georgia Regents University, United States of America

## Abstract

Periosteum provides a major source of mesenchymal progenitor cells for bone fracture repair. Combining cell-specific targeted *Cox-2* gene deletion approaches with *in vitro* analyses of the differentiation of periosteum-derived mesenchymal progenitor cells (PDMPCs), here we demonstrate a spatial and temporal role for Cox-2 function in the modulation of osteogenic and chondrogenic differentiation of periosteal progenitors in fracture repair. *Prx1Cre*-targeted *Cox-2* gene deletion in mesenchyme resulted in marked reduction of intramembraneous and endochondral bone repair, leading to accumulation of poorly differentiated mesenchyme and immature cartilage in periosteal callus. In contrast, *Col2Cre*-targeted *Cox-2* gene deletion in cartilage resulted in a deficiency primarily in cartilage conversion into bone. Further cell culture analyses using *Cox-2* deficient PDMPCs demonstrated reduced osteogenic differentiation in monolayer cultures, blocked chondrocyte differentiation and hypertrophy in high density micromass cultures. Gene expression microarray analyses demonstrated downregulation of a key set of genes associated with bone/cartilage formation and remodeling, namely *Sox9*, *Runx2*, *Osx*, *MMP9*, *VDR* and *RANKL*. Pathway analyses demonstrated dysregulation of the HIF-1, PI3K-AKT and Wnt pathways in Cox-2 deficient cells. Collectively, our data highlight a crucial role for Cox-2 from cells of mesenchymal lineages in modulating key pathways that control periosteal progenitor cell growth, differentiation, and angiogenesis in fracture repair.

## Introduction

Fracture healing is a unique postnatal bone regeneration process that occurs as a cascade of well-orchestrated biological events leading to the restoration of bone tissue. Fracture healing requires the formation of an external bone callus, which is initiated primarily by the progenitor cells residing in the periosteum [1]–[4]. Analogous to embryonic skeletal development, periosteum-initiated fracture repair implicates endochondral and intramembranous bone formation, which proceed in a sequential and organized manner [5], [6]. While adult bone repair recapitulates some essential regulatory mechanisms that occur in early skeletal development, repair is a unique bone morphogenetic process, orchestrated by an ensemble of genes distinct from early skeletal development [7]. Due to an inability to directly target the periosteum, the molecular mechanisms and the implicated molecular pathway(s) that control the differentiation program of periosteal mesenchymal progenitor cells in bone fracture repair remains poorly understood. Identifying the critical genes in periosteum-initiated bone repair, establishing their spatiotemporal expression, and elucidating their integrated roles will be essential to understand bone regeneration and to develop useful therapeutics to improve skeletal repair and reconstruction.

Cox-2 is the inducible isoform of cyclooxygenase, the enzyme responsible for a major control step in prostanoid biosynthesis pathway. Cox-2 plays an important role in cancer biology, in vascular pathophysiology, and in a variety of inflammatory disorders [8], [9]. Global deletion of the *Cox-2* gene in mice does not affect overall skeletal development [10], [11]. However, global absence of Cox-2 markedly impairs fracture healing [12], [13]. An important role for Cox-2 in fracture healing has been shown in aged animals. Older mice have a marked reduction of *Cox-2* expression in the fracture callus, exhibiting delayed neovascularization and endochondral bone formation [14]. *In-situ* hybridization analyses demonstrate that *Cox-2* expression peaks at the early stage of intramembranous and endochondral stage of bone healing [15]. Elevated *Cox-2* expression is detected in chondroprogenitors and proliferating chondrocytes at days 5 and 7 post-fracture. Cox-2 expression is subsequently reduced in hypertrophic chondrocytes during the remodeling phase of healing, suggesting that Cox-2 expression is tightly regulated during fracture repair. In contrast to loss of Cox-2 function, Cox-2 gain of function by overexpression at the healing site accelerates fracture healing in animal models [16].

While an essential role of Cox-2 in fracture repair has been established, targeted tissues and implicated pathways remain unclear. Here we utilize two tissue-specific promoter driven-Cre transgenic mouse lines to delete the *Cox-2* gene in limb mesenchymal lineages (with Prx1Cre) and in chondrocytes (with Col2Cre), respectively. To determine the mechanistic involvement of Cox-2 in control of osteogenic and chondrogenic differentiation of periosteal mesenchymal progenitors, we further performed differentiation and gene expression profile analysis using periosteum-derived mesenchymal progenitor cells (PDMPCs) isolated from the healing periosteum of the mutant and control mice [17], [18]. Our study established a critical role for Cox-2 in the differentiation paradigm of periosteal mesenchymal progenitor cell in fracture repair, underscoring the importance of spatial and temporal regulation of Cox-2 in bone repair and regeneration.

## Materials and Methods

### Animal models

To determine the gene recombination efficiency of Prx1Cre and Col2Cre lines in fracture callus, *Col2Cre; RosaR* and *Prx1Cre; RosaR* mice were generated and characterized for beta-galactosidase expression. Cox-2 conditional deletion (*Cox-2^f^* ^/f^) mice [19] were crossed with *Prx1-1Cre* or *Col2Cre* transgenic mice to produce *Cox-2^f/f^; Prx1Cre* and *Cox-2^f/f^; Col2Cre* mice. All studies and procedures were approved by the Institutional Animal Care and Use Committee at the University of Rochester. Littermates were used for analysis.

### Fracture healing model

Closed stabilized femoral fractures were created in two month-old mice [14], [15]. Mice were anesthetized with a mix of Ketamine and Xylazine. The skin and the underlying soft tissues over the knee were incised lateral to the patellar tendon. The tendon was displaced medially, and a small hole was drilled into the distal femur using a 26-gauge needle. A stylus pin from a 25G Type spinal needle (BD Medical Systems, Franklin Lakes, NJ) was inserted into the intramedullary canal and clipped. The wound was closed by suturing. Fractures were created at the diaphyseal region of mouse femurs using a three-point bending Einhorn device, as previously described [20]. Fracture healing was examined in gender and age-matched littermates. *Cox-2^f/f^; Col2Cre* and *Cox-2^f/f^; Prx1Cre* were compared with their respective Cre-negative *Cox-2* ^f/f^ littermate controls for analyses.

### Micro-CT Imaging Analyses

Femurs were harvested at indicated time points and scanned using a Viva micro-CT system (Scanco Medical, Switzerland) at a voxel size of 10.5 µm to image bone. New bone formation was measured as previously described [21]. The threshold was chosen using 2D evaluation of several slices in the transverse anatomical plane. In this way, mineralized callus was identified while surrounding soft tissue was excluded. An average threshold of 220 was optimal and was used uniformly for all samples. Each sample was contoured around the external callus and along the edge of the cortical bone, excluding the marrow cavity. New bone volume was measured on the surface of fracture samples as previously described [15]. Gender and age matched littermates were used for analyses. Indices of cortical bone morphology from the diaphyseal tibia were assessed by micro-CT imaging as described previously [11]. Cortical bone morphology in male and female mice were analyzed separately and presented as gender-matched groups as indicated.

### Histology and histomorphometric Analyses

Fractured femurs were harvested and processed for histological analyses as previously described [12]. Femurs were disarticulated from the hip and trimmed to remove excess muscle and skin. Specimens were stored in 10% neutral buffered formalin for 3 days. The tissues were infiltrated and embedded in paraffin. Sections were prepared and stained with Alcian blue/Hematoxylin as previously described [12]. Histomorphometric analyses were performed using Osteometrics software to determine the area of bone, cartilage, and mesenchyme (a subtraction of total callus from bone and cartilage tissue) by manual tracing. Pre-existing cortical bone was excluded from the analyses. The percent areas of bone, cartilage, and mesenchyme were used to illustrate the composition of the fracture callus. At least three nonconsecutive sections from each sample were used for histomorphometric analyses. The means of ten samples from each group were used for statistical analyses.

### Isolation of periosteum-derived mesenchymal progenitors (PDMPCs) from autograft periosteum

We have previously devised a method which allows isolation of sufficient numbers of periosteum-derived mesenchymal progenitors (PDMPCs) from day 5 periosteum callus of autografts to perform *in vitro* differentiation analyses [17], [18]. Briefly, autograft transplantations were performed in *Cox-2^f/f^; Prx1Cre* mice and their Cre-negative control mice. Mice were anesthetized by peritoneal injection of a mix of Ketamine and Xylazine. A 7–8 mm long incision was made in hind limb, and the mid-shaft femur was exposed by blunt dissection of muscles without disturbing the periosteum. A 4-mm mid-diaphyseal segment was removed from the femur of the donor mice using a sharp diamond-cutting wheel attached to a cordless dremel. The same 4 mm cortical bone graft was then inserted back into the segmental defect and stabilized by a 22-gauge metal pin placed through the intramedullary marrow cavity (autograft transplantation). Donor bone autografts were collected at day 5 post-transplantation. Bone marrow inside the bone graft was removed and discarded by repeated flushing of the marrow cavities with serum-free α-MEM medium. Periosteum tissues were scraped off and pooled in a Petri dish. After digestion with Collagenase D (Roche Applied Science, Indianapolis, IN) at a concentration of 1 mg/ml for 1 hour, cells released from periosteal tissues were pooled and cultured in α-MEM medium containing 1% penicillin and streptomycin, 1% glutamine, and 20% fetal bovine serum (FBS). Once confluent, cells were trypsinized and further expanded in α-MEM medium containing 10% FBS. Periosteal cells from second and third passage were collected and used for all experiments.

For osteogenesis assays, cells isolated from Cox-2^f/f^; Prx1Cre mice and their Cre-negative control mice were cultured as monolayers in fresh α-MEM media containing 1% penicillin and streptomycin, 1% glutamine, and 10% fetal bovine serum (FBS). Since PDMPCs can spontaneously differentiated into osteoblastic cells in regular media following 7 day culture, the basal level of differentiation was examined in control and KO cells in regular media. To examine osteogenic differentiation in response to BMP-2, identical amount of BMP-2 (100 ng/ml) was added to the control and KO culture. Cells were harvested on day 7 for Alkaline Phosphatase staining (ALP) staining and RNA analyses as previously described [17], [18]. For chondrogenesis assays, 2×10^5^ cells per well were seeded as micromass in a 24-well plate and cultured in DMEM media with 10% fetal bovine serum with or without identical amount of BMP-2 (100 ng/ml). Cell pellets were harvested on day 1 and 7 for Alcian Blue staining and gene expression analyses.

### Real-Time PCR Analyses

Total RNA was prepared using a Qiagen RNA extraction kit. Exactly 0.5 µg of mRNA from 4 different samples was pooled and reverse transcribed to make single-strand cDNA, using a commercial first strand cDNA synthesis kit (Invitrogen). Quantitative RT-PCR reaction was performed using SyberGreen (ABgene, Rochester, NY) in a RotorGene real time PCR machine (Corbett Research, Carlsbad, CA). All genes were compared to a standard β-actin control. Data were assessed quantitatively using analysis of variance, comparing relative levels of transcript expression as a function of time. All primers used for the assessment can be found in previous publications [15], [17], [18] or listed in Table S2. Data are expressed as the means ± SEM. Statistical significance between experimental groups was determined using one-way ANOVA and a Tukey's posthoc test (GraphPad Prism, San Diego, CA). A P value <0.05 was considered statistically significant.

### Western blot analyses

Cells were lysed in Golden lysis buffer supplemented with protease inhibitor (Roche Applied Science). The protein extracts (10 µg) were separated using NuPAGE BisTris gels (Invitrogen). Gels were transferred to a polyvinylidene difluoride membrane (PerkinElmer Life Sciences Waltham, MA) and probed with anti–Cox-2 (Cayman Chemical Inc, Ann Arbor, MI) and anti–β-actin monoclonal antibody (Sigma, St. Louis, MO).

### Microarray analyses

For microarray analysis, a total of 24 RNA samples in 8 indicated groups (n = 3 per group) were prepared from micromass cultures of Cox-2^f/f^; Prx1Cre or Cox-2^f/f^ PDPMCs, with or without BMP-2 treatment, at day 1 and day 7. Total RNA from each sample was isolated using an RNeasy Mini extraction kit. RNA quality and purity were determined using a NanoDrop ND-1000 spectrophotometer (NanoDrop Technologies, Wilmington, DE, USA). RNA integrity was determined by the Agilent 2100 bioanalyser (Agilent Technologies, Palo Alto, CA, USA). Whole mouse gene expression microarrays (Ilumina, BD-202-0202), containing over 25,600 unique probes and over 19,100 unique genes, were used to detect the gene expression profile each sample. The raw data obtained from all 24 samples were normalized by applying a background correction (using the ‘normexp’ algorithm) followed by normalization of intensity distributions within and between arrays (using the ‘quantile’ algorithm). The resulting data were imported into Partek Genomics Suite (Partek Inc., St. Louis, MO) and log2 transformed for statistical processing and hierarchical clustering analyses. Differential gene expression and hierarchical clustering were generated from comparison between 8 different groups, using one-way ANOVA. Differentially expressed genes were selected with a p value less than 0.01 and a fold of change of more than 2 when comparing between groups. Heat maps were generated by Partek Genomics Suite software. All raw and processed data files have been deposited in the National Center for Biotechnology Information Gene Expression Omnibus dataset.

Biological processes, functional classifications and gene annotations were analyzed using Partek Genomics Suite associated with Kyoto Encyclopedia of Genes and Genomes (KEGG) pathway database (updated December 2013), as well as database for Annotation, Visualization and Integrated Discovery (DAVID) (http://david.abcc.ncifcrf.gov). To identify biological processes with significant enrichment, the distribution of genes from our data was compared with a reference annotation gene list for each gene ontology (GO) category. Fisher exact P values were used for gene enrichment analysis. The value ranges from 0 to 1, where value equal to zero represents perfect enrichment. P value less than or equal to 0.05 is considered significantly enriched in the annotation categories.

## Results

### Targeted Cox-2 gene deletion in cartilage or mesenchyme results in impaired fracture healing

**Figure 1:**
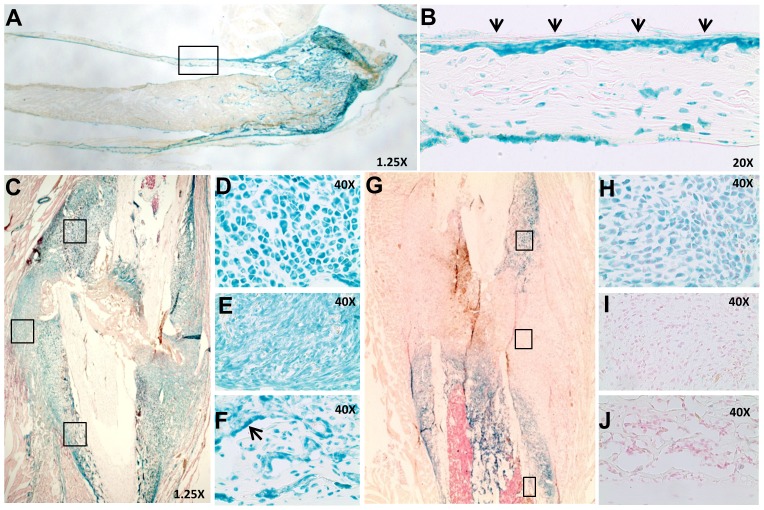
Efficient Prx1Cre- and Col2Cre-mediated targeted gene recombination in fracture callus. A tissue section from long bone of *Prx1Cre; RosaR* mice show intense LacZ staining in bone and cartilage, but not in bone marrow (A). The boxed region in A, shown at a higher magnification (20×), demonstrates LacZ staining in periosteum (arrows in B). Fracture callus at day 7 from *Prx1Cre; RosaR* shows intense LacZ staining throughout the callus region at the cortical bone junction (C). Boxed regions in C (from top to bottom), shown at a higher magnification (20×), illustrate effective gene recombination in chondrocytes (D), mesenchyme (E) and osteoblasts (arrows in F). *Col2Cre; RosaR* fracture callus at day 5 shows effective gene recombination in chondroprogenitors and chondrocytes, but not mesenchymal cells (G). Higher magnification images (20×) in the boxed region (from top to bottom) show positive LacZ staining in chondrocytes (H) but not in mesenchyme cells (I) or osteoblasts (J).

To establish Cre-recombinase mediated gene targeting efficiency and specificity, femoral fractures were created in two-month-old *Prx1Cre; RosaR* and *Col2Cre; RosaR* mice. Prior to fracture in intact bone, intense LacZ staining was identified in all limb mesenchymal lineages, including strong staining in the periosteum of Prx1Cre; RosaR mice (Fig. 1A&B). Bone marrow and muscle were largely negative for LacZ staining. Following fracture, LacZ staining was observed in mesenchyme, chondrocytes and osteoblasts throughout the fracture callus in Prx1Cre; RosaR mice at day 7, indicating efficient gene recombination in all limb mesenchymal lineages (Fig. 1C–F). In *Col2Cre; RosaR* fracture callus, strong LacZ staining was observed as early as day 5 post-fracture, primarily in chondrocytes along the periosteal surface and within the bone marrow cavity, where endochondral bone formation takes place (Fig. 1G&H). Mesenchymal progenitors (Fig. 1I) at the fracture junctions and osteoblasts (Fig. 1J) at the distal flanking region of the callus remained negative for LacZ staining.

**Figure 2:**
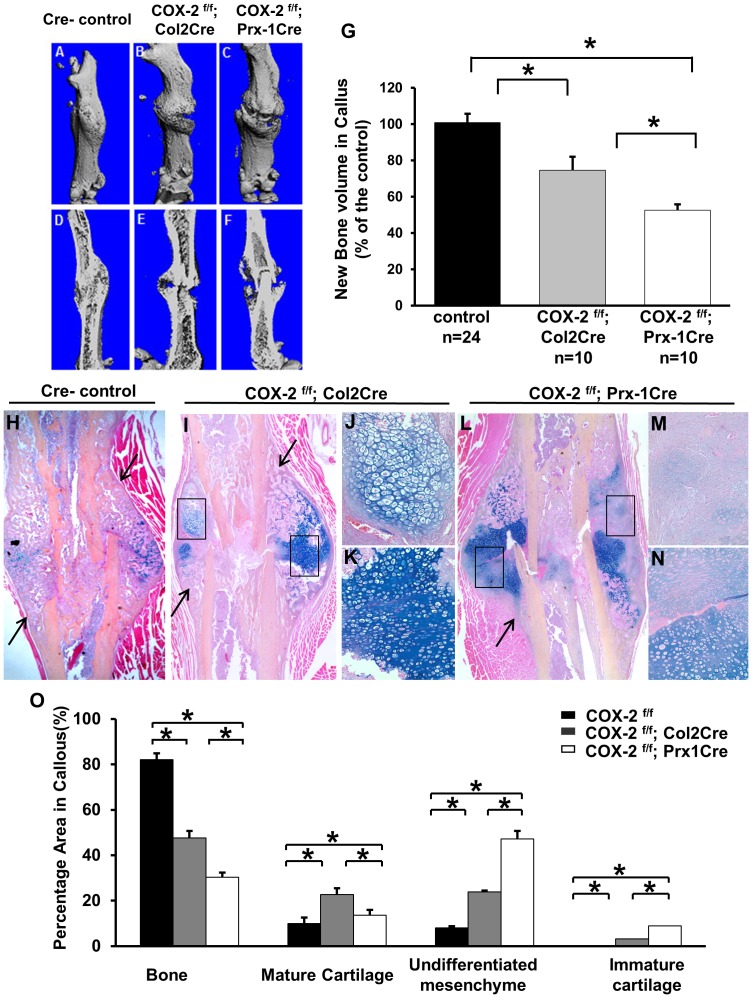
Targeted *Cox-2* gene deletion via Prx1Cre and Col2Cre impaired fracture healing. Representative micro-CT images of fracture callus at day 14 post-fracture in control *Cox-2^f/f^* (A and D), *Cox-2^f/f^; Col2Cre* (B and E) and *Cox-2^f/f^; Prx1Cre* (C and F). Volumetric analyses demonstrate marked reduction of new bone formation in *Cox-2^f/f^; Prx1Cre* and *Cox-2^f/f^; Col2Cre* fracture callus (G). Representative histology sections of fracture callus at day 14 from control *Cox-2^f/f^* (H), *Cox-2^f/f^; Col2Cre* (I–K), and *Cox-2^f/f^; Prx1Cre* (L–N) mice. Boxed regions in I show presence of mature (J) and under-differentiated chondrocytes (K) in *Cox-2^f/f^; Col2Cre* fracture callus. Boxed regions in L show poorly differentiated mesenchyme (M) and immature cartilage (N) in *Cox-2^f/f^; Prx1Cre* fracture callus. Arrows indicate regions of intramembraneous bone formation in H, I and L. Quantitative histomorphometric analyses show the composition of bone, cartilage and mesenchyme tissue in periosteal callus (K). Data are presented as means ± SEM, * p<0.05. n = 10.

Long bone length and cortical bone morphology were examined in *Cox-2^f/f^; Prx1Cre* mice and their littermate controls. No significant differences in long bone length or cortical bone thickness could be determined between *Cox-2^f/f^* and *Cox-2^f/f^; Prx1Cre* mice (Fig. S1), consistent with our previous findings in global Cox-2^−/−^ mice [11]. Fracture healing was examined in both *Cox-2^f/f^; Prx1Cre* and *Cox-2^f/f^; Col2Cre* mice, along with gender and age-matched control Cre-negative *Cox-2^f/f^* mice. Micro-CT analyses showed delayed bony union in both *Cox-2^f/f^; Prx1Cre* and *Cox-2^f/f^; Col2Cre* mice at day 14 post-fracture (Fig. 2A–F). Quantitative and volumetric analyses demonstrated a 47% and a 25% reduction of new bone callus in *Cox-2^f/f^; Prx1Cre* and *Cox-2^f/f^; Col2Cre* mice, respectively (Fig. 2G). Evaluation of new bone callus from individual micro-CT-images suggested that 80% of Cre negative mice demonstrated mature union with formation of a complete bridging callus on day 14. In contrast, only 10% of *Cox-2^f/f^; Col2Cre* mice showed mature union and none of the *Cox-2^f/f^; Prx1Cre* mice showed any evidence of bony union at day 14 post-fracture.

Histologic analyses showed mature bridging callus at day 14 in the *Cox-2^f/f^* control mice of both groups, with only a small amount of residue cartilage present in the callus (Fig. 2H). In contrast, substantial amounts of cartilaginous tissue remained in the fracture callus of *Cox-2^f/f^; Col2Cre* mice at day 14 (Fig. 2I). Cartilage conversion into bone was markedly reduced, yet intramembranous bone formation flanking the cartilaginous tissue (arrows in Fig. 2I) remained largely intact in these mice. Careful examination of the cartilaginous tissue showed that they were mostly mature chondrocytes (Fig. 2J) or less differentiated chondrocytes (Fig. 2K). In *Cox-2^f/f^; Prx1Cre* fracture callus, where *Cox-2* is deleted in mesenchymal progenitors, severe reduction of bone formation at the periosteal sites was evident (Fig. 2L). Extensive mesenchyme (Fig. 2M) and poorly differentiated cartilage tissue (Fig. 2N) were observed throughout the fracture callus. Histomorphometric analyses revealed marked differences in callus composition among the three groups of mice at day 14 post-fracture (Fig. 2K). Compared to the Cre-negative controls which contained 8% mesenchyme, 9.9% mature cartilage and 0% immature cartilage in the fracture callus, *Cox-2^f/f^; Prx1Cre* mice had an average of 52% mesenchyme and 12% immature cartilage in the fracture callus (Fig. 2K, open bar, n = 10, p<0.05). As a result, the percentage of new bone formation in *Cox-2^f/f^; Prx1Cre* mice was reduced by nearly 3-fold as compared to their Cre-negative controls. In *Cox-2^f/f^; Col2Cre* callus, the percentage area of mesenchyme was increased to 25% of the total callus. However, unlike *Cox-2^f/f^; Prx1Cre* callus, which was primarily occupied by mesenchyme and poorly differentiated cartilage, the *Cox-2^f/f^; Col2Cre* callus had an average of 23% mature cartilage and 3% immature cartilage at the cortical bone junctions, leading to a 1.8-fold reduction of new bone formation in the fracture callus (Fig. 2K, gray bar, n = 10, p<0.05).

### Cox-2 deficient periosteal progenitors exhibit impaired osteogenic and chondrogenic differentiation in cell culture

**Figure 3:**
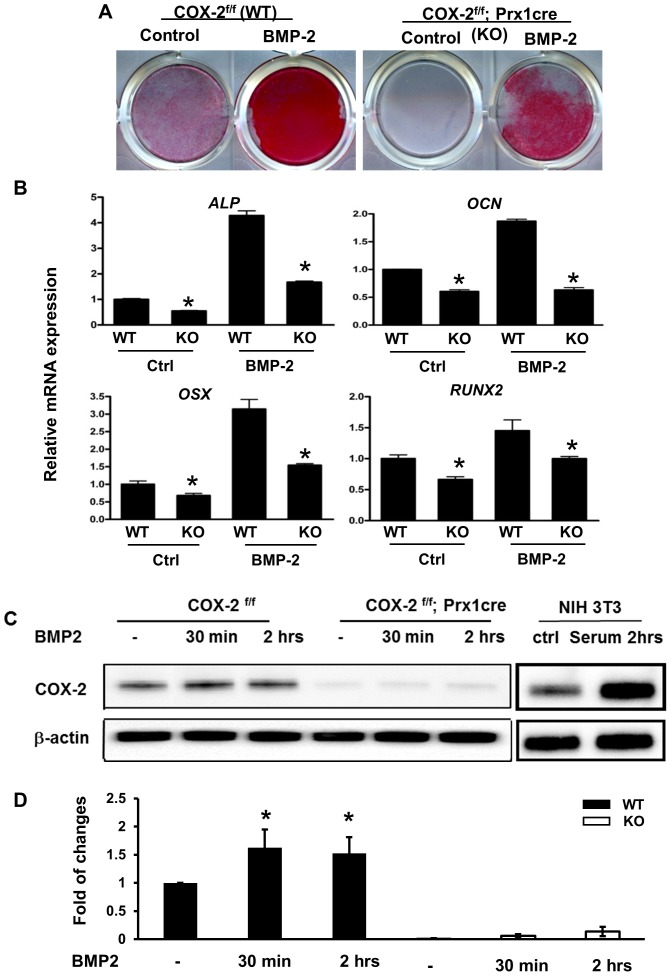
Osteogenic differentiation was impaired in Cox-2 deficient PDMPCs. Periosteal progenitors were isolated from *Cox-2^f/f^* (WT) and *Cox-2^f/f^; Prx1Cre* (KO) mice. Monolayer cultures demonstrate reduced differentiation of *Cox-2* deficient cells, both under basal conditions and in response to BMP-2 stimulation, as evidenced by reduced ALP staining (A) and decreased osteogenic gene expression at day 7 (B). * indicates p<0.05, as compared to the control. Western blot analyses demonstrate a modest induction of Cox-2 protein in WT cells and ablation of Cox-2 protein in Cox-2 deficient cells (C). Quantification of western blot analyses from three separate experiments shows induction of Cox-2 protein in WT cells and near absence of Cox-2 protein in the Prx-1Cre-mediated conditional KO cells (*, p<0.05).

To further understand the role of Cox-2 in periosteum-mediated repair, PDMPCs were isolated from the periosteum of *Cox-2^f/f^; Prx1Cre* mice and their Cre-negative littermate controls. Cox-2-deficient PDMPCs exhibited reduced ALP staining both at the basal level and following BMP-2 stimulation (Fig. 3A). Prx1Cre-mediated *Cox-2* gene deletion also markedly reduced *ALP*, *RUNX2*, *OSX* and *OCN* gene expression, both in untreated cultures and upon BMP-2 treatment (Fig. 3B). Western blot analyses demonstrated a modest, but statistically significant, induction of Cox-2 protein by BMP-2 treatment in control cells and the absence of Cox-2 protein in PDMPCs isolated from *Cox-2^f/f^; Prx1Cre* mice (Fig. 3C). Quantification of Western blot data from three experiments demonstrates an average of ∼1.5 fold induction of Cox-2 by BMP-2 in monolayer cultures from COX-2^ff^ (wild type) mice and ∼95% reduction of Cox-2 protein in the Cox-2^f/f^; Prx1Cre cells (Fig. 3D).

**Figure 4:**
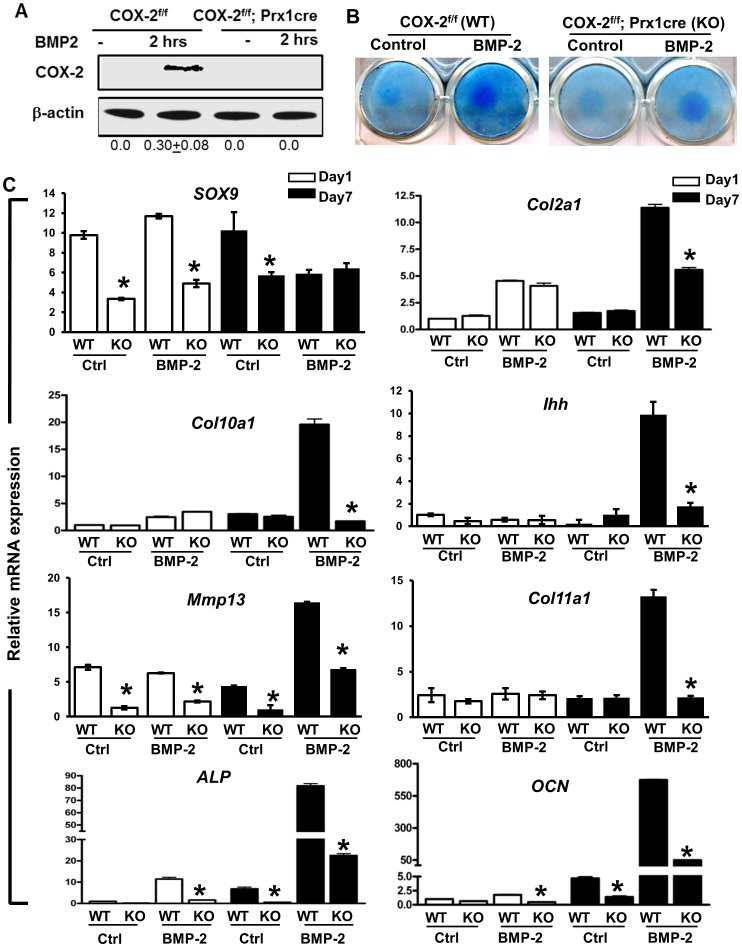
Chondrogenic differentiation was impaired in periosteal progenitors from mice with a targeted *Cox-2* gene deletion. Periosteal mesenchymal progenitors were isolated from *Cox-2^f/f^* (WT) and *Cox-2^f/f^; Prx1Cre* (KO) periosteum. Western blot analyses demonstrated marked Cox-2 induction by BMP-2 in WT cells and absence of Cox-2 protein in Cox-2 deficient cells. Numbers at the bottom of the image show normalized density ratio of each lane in western blot analyses (A). Micromass cultures demonstrate impaired chondrogenesis and chondrocyte differentiation in KO cells at day 1 and day 7, as indicated by Alcian Blue staining (B) and Real Time PCR analyses of genes associated with chondrocyte differentiation and bone formation (C). * p<0.05, as compared to the control.

Chondrogenesis and chondrocyte differentiation were examined in PDMPC micromass cultures (Fig. 4). Two time points that reflect chondrogenesis onset (day 1) and chondrocyte maturation (day7) were examined [22], [23], in the presence and absence of BMP-2 treatment (n = 3 per group). In contrast to monolayer culture, Western blot analyses demonstrated that Cox-2 protein was markedly induced by BMP-2 in the high-density micromass cultures (Fig. 4A), suggesting Cox-2 as a BMP-2 responsive gene in chondrogenic conditions. The induction of Cox-2 expression by BMP-2 was further confirmed by RT-PCR analyses (Fig. S2); these data demonstrate robust induction of *Cox-2* mRNA expression in both day 1 and day 7 BMP-2 treated cultures. Prx1Cre-mediated *Cox-2* gene deletion decreased chondrogenesis and chondrocyte differentiation induced by BMP-2, as evidenced by reduced Alcian Blue staining (Fig. 4B), suppressed *SOX-9* expression at day 1 and further reduced expression of a set of chondrocyte marker gene expression at day 7 (Fig. 4C). Of note is that *Cox-2* gene deletion reduced BMP-2-induced *Col2a1* expression by 50%, but blocked the expression of BMP-2-induced chondrocyte maturation genes, namely *Col10a1*, *Ihh*, *MMP13 and Col11a1* at day 7 (Fig. 3C), suggesting that mesenchymal cell-specific Cox-2 expression is required for both chondrogenesis and chondrocyte maturation and hypertrophy. Consistently, *ALP* and *OCN*, the bone marker genes associated with endochondral ossification, were similarly reduced in BMP-2-treated culture at day 7, demonstrating a key role of Cox-2 in mesenchymal differentiation.

To obtain a deeper understanding of the molecular regulation of osteogenic and chondrogenic differentiation of PDMPCs, gene expression microarray analyses were performed. These differential gene profiling studies are directed at identifying Cox-2 mediated differences in gene expression in micromass cultures of *Cox-2^f/f^; Prx1Cre* and *Cox-2^f/f^* PDMPCs. In the absence of BMP-2 at day 1, 143 genes were found to be suppressed by 2-fold or more in the Cox-2 deficient cells as compared to the control PDMPCs. Biological GO enrichment analyses show functional cluster categories of genes involved in bone development and ossification process (11 genes, p<4.0E-5), immune/inflammatory response (21 genes, p<8.2E-5), growth factor activity (7 genes, p<3.3E-4), Wnt pathway (6 genes, p<0.001) and morphogenesis of a branching structure (7 genes, p<0.005) (Table S1). Several known key regulators associated with bone/cartilage ossification and remodeling were significantly suppressed in the Cox-2 deficient cells, namely *Sox9* (2.7-fold), *Sp7 (OSX)* (2.2-fold), *MMP13* (4.5-fold), *MMP9* (4.8-fold), *RANKL (3.7-fold) and Vitamin D receptor (VDR)* (4.8-fold), suggesting reduced osteogenic/chondrogenic potential and bone/cartilage remodeling activity in Cox-2 deficient cells. Following micromass culture for 7 days, PDMPCs underwent spontaneous differentiation to induce a series of genes critical for bone formation, namely *SP7* (5.1-fold), *BMP4* (23.5-fold), and *FGFR3* (4.3-fold), and *Osteocalcin* (4-fold). Among the genes associated with bone/cartilage development and ossification, we identified 25 genes that were significantly suppressed at day 7 in the Cox-2 deficient cells (Fig. S3B), providing further evidence to show the disruption of bone morphogenetic pathway in micromass culture as a consequence of targeted *Cox-2* gene deletion in PDMPCs.

**Figure 5:**
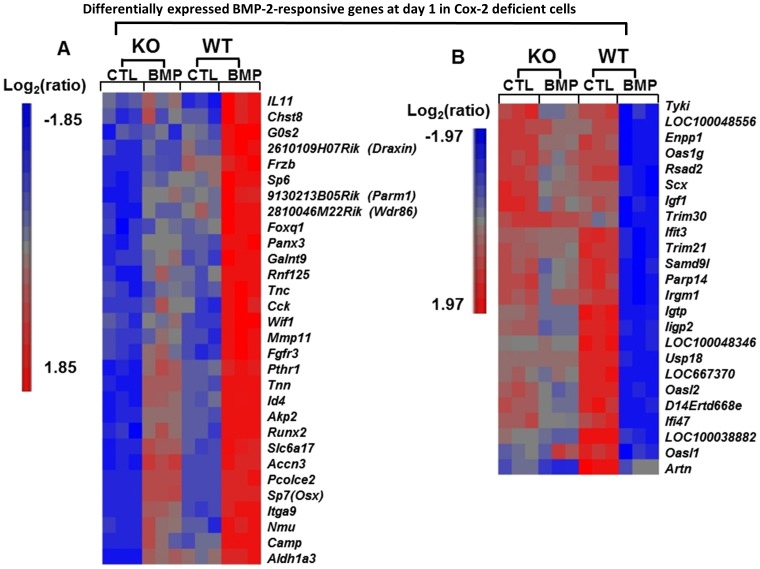
Differentially down- and up-regulated genes in *Cox-2^f/f^* and *Cox-2^f/f^; Prx1Cre* PDMPCs in response to BMP-2. Heat map showing differential expression of 30 BMP-2-upregulated genes (A) and 24 BMP-2-downregulated genes (B) in Cox-2^f/f^ (WT) in micromass cultures at day 1 and their corresponding expression levels in BMP-2 treated *Cox-2^f/f^; Prx1Cre* (KO) micromass cultures. Each column shows the relative gene expression of a sample for the indicated pathway-associated genes. Gene up-regulation is presented in red and gene down-regulation is in blue.

To further identify BMP responsive genes whose expression is mediated by COX-2, we separately analyzed the gene profiling in control and Cox-2 deficient PDMPCs at day 1 and day 7 following BMP-2 treatment. At day 1, among 193 BMP-2-responsive genes whose expression were changed by 2-fold or more in control *Cox-2^f/f^* cells, 30 of these genes had significantly suppressed expression in Cox-2 deficient cells in response to BMP-2. These 30 genes are known to be involved in regulation of cellular proliferation and differentiation and in developmental process associated with bone/cartilage formation (Fig. 5A). Among genes that were markedly down-regulated by one day of exposure to BMP-2 in *Cox-2^f/f^* (WT) cells, 20 of those genes were less down-regulated by BMP-2 in *Cox-2* deficient cells (Fig. 5B). These genes were functionally mapped to annotation categories of immune process and response to stress.

Markedly differences in gene expression profiles were identified in samples treated with BMP-2 at day 7. In control *Cox-2^f/f^* cells, a total of 1183 unique genes were identified that exhibited a change of 2-fold or more following BMP-2 treatment (Fig. S4A). Among BMP-2 upregulated genes, 447 unique transcripts had significantly reduced expression in BMP-2-treated *Cox-2* deficient cells. Gene ontology (GO) enrichment analyses using DAVID and Partek-associated software annotated these 449 differentially expressed genes into several major categories, including bone/cartilage development and ossification (p = 2.0E-8), glycolysis/gluconeogenesis (p = 7.1E-7), extracellular matrix (p = 3.0E-10), angiogenesis and vessel development (p<0.01) (Table 1). Among genes down-regulated by BMP-2 in *Cox-2^f/f^* WT cells, 208 genes showed less suppressed expression by BMP-2 in *Cox-2* deficient cells. These genes were functionally mapped to annotation categories of immune system response, leukocytes and osteoclast differentiation, biological adhesion, and angiogenesis (Table 1). The marked differences in gene expression profile between Cox-2 deficient cells and control PDMPCs in response to BMP-2 strongly suggest Cox-2 as one of the important downstream mediators of BMP-2.

**Table 1:** Go classification of the differentially expressed genes in Cox-2 deficient cells at day 7 in response to BMP-2.

Functional classification	Fisher exactp value	Genes included in the group
**Upregulated by BMP-2 in WT, significantly suppressed in KO (447 genes)**		
Bone/cartilage development process (ossification)	2.0E-8	39
Glycolysis/gluconeogenesis	7.1E-7	14
Extracellular matrix	3.0E-10	27
Angiogenesis and vessel development	<0.01	24
**Down-regulated by BMP-2 in WT, significantly less regulated in KO (208 genes)**		
Immune system response	3.0E-5	42
Leukocytes and osteoclasts differentiation	9.6E-4	13
Biological adhesion	6.3E-3	15
angiogenesis	<0.01	14

The table lists major functional categories enriched by DAVID using differentially expressed, BMP-2 up-regulated or down-regulated genes following seven days of culture in the presence or absence of BMP-2. Fisher exact P values for the gene-enrichment categories were generated by DAVID. “Genes included in the group” indicate the number of genes enriched for that category from the input gene list.

### Enriched biological pathway analyses demonstrate dysregulation of the HIF1, PI3K-AKT and Wnt pathways in Cox-2 deficient PDMPCs

**Figure 6:**
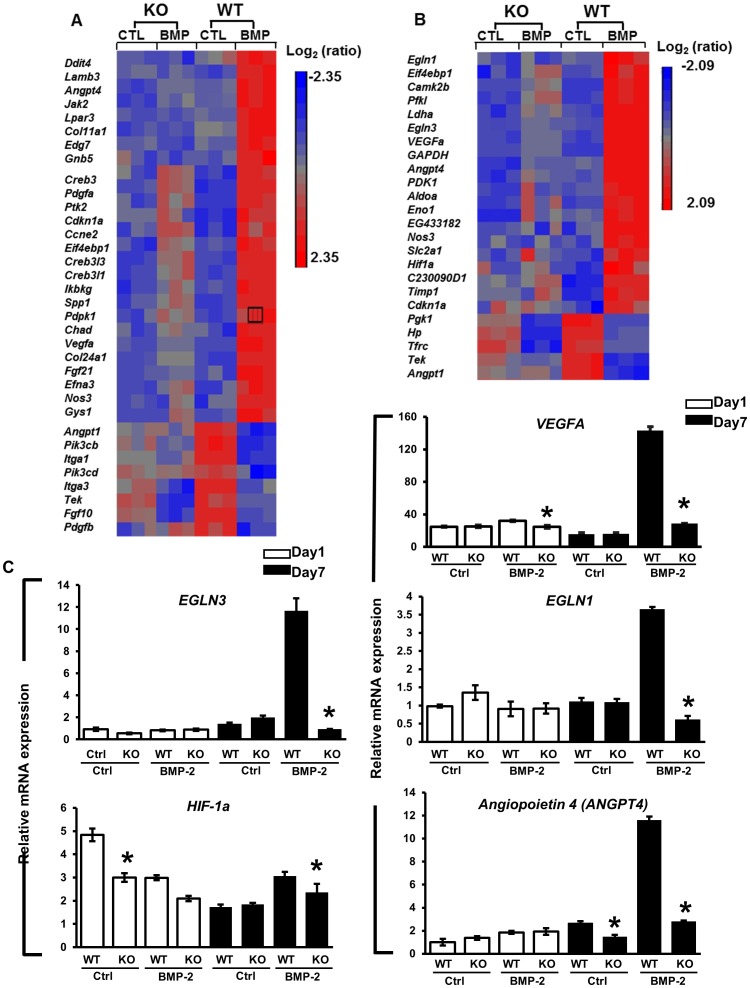
Dysregulation of the PI3K/AKT and HIF-1pathways in Cox-2 deficient PDMPCs. Heat maps showing differentially expressed genes associated with the PI3K/AKT (A) and the HIF-1 pathway (B) in micromass cultures in the presence and absence of BMP-2 for seven days. Each column shows the relative gene expression of a sample for the indicated pathway-associated genes. RT-PCR analyses further quantitate the values for the key genes in the HIF-1 pathway at day 1 (white bars) and day 7 (black bars) (C), namely *VEGFA*, *EGLN1*, *EGLN3*, *HIF-1a* and *ANGPT4*. * p<0.05, as compared to the control.

The genes differentially expressed in *Cox-2^f/f^ and Cox-2^f/f^; Prx1Cre* PDMPCs at day 1 and day 7 in the presence and absence of BMP-2 were further analyzed using the KEGG pathway database available in the Partek Genomic Suite. The main signaling pathways dysregulated by the absence of Cox-2 are the PI3K-AKT, HIF-1 and Wnt pathways. At day 1, genes annotated to the PI3K-AKT pathway, whose expression were suppressed in *Cox-2* deficient cells in the presence of BMP-2, include *FGFR2* (1.4-fold, p<0.01), *FGFR3* (2.6-fold, p<0.01), *Itga9 (1.6fold*, *p<0.01)*, *Tnc (2.4-fold*, *p<0.01)*, *Tnn (2.2fold*, *p<0.01)*. At day 7, 34 of the annotated PI3K-AKT pathway genes had significantly altered expression in *Cox-2* deficient cells; eight of these genes were down-regulated, relative to wild-type cells, in the absence of any treatment and 26 of these genes were down-regulated, relative to wild type cells, following BMP-2 treatment (Fig. 6, top panel A).

Additional remarkable changes identified in Cox-2 deficient PDMPCs were the altered gene expressions annotated to the hypoxia inducible factor 1 (HIF-1) pathway. Among 41 HIF-1 pathway genes identified from control *Cox-2^f/f^ c*ells at day 7, 24 genes showed altered expression in the Cox-2 deficient cells (Fig. 6. top panel B). Real-time PCR analyses further confirmed the suppressed expression of several key genes of the HIF-1 pathway, namely *EGLN1*, *EGLN3*, *VEGFA*, *ANGPT4 and HIF-1a* in Cox-2 deficient cells day 7 (Fig. 6C). These data indicate strongly a key role for Cox-2 in modulating HIF-1 pathway activation in PDMPCs.

**Figure 7:**
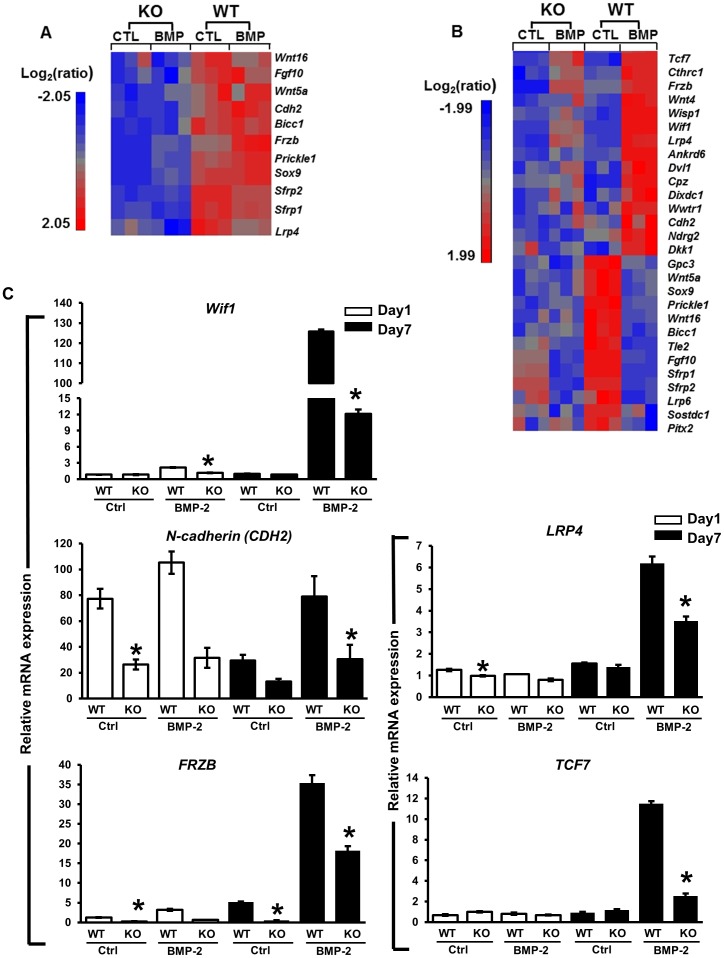
Deferentially expressed genes associated with the Wnt pathway in Cox-2^f/f^ and Cox-2^f/f^; Prx1Cre PDMPCs. Heat maps showing differentially expressed genes associated with the Wnt pathway at day 1 (A) and day 7 (B) micromass cultures. Each column shows the relative gene expression of a sample for the indicated Wnt pathway-associated genes. RT-PCR demonstrates expression of key genes in the Wnt pathway at day 1 (white bars) and day 7 (black bars) in cells with a targeted *Cox-2* gene deletion and in their littermate controls (C), namely *Wif1*, *N-cadherin (CDH2)*, *LRP4*, *FRZB*, and *TCF7*. * p<0.05, as compared to the control.

Wnt pathway genes were also enriched among the genes differentially expressed between *Cox-2^f/f^ and Cox-2^f/f^; Prx1Cre* PDMPCs at days 1 and 7. Several well-documented Wnt pathway inhibitory genes involved in bone metabolism were significantly suppressed at basal level in Cox-2^f/f^; Prx1Cre PDMPCs at day 1 in micromass culture, including *Prickle1*, *Cdh2*, *Frzb*, *Sfrp1 and 2* (Fig. 7A). At day 7, 28 Wnt pathway-associated genes were suppressed in untreated cultures or following BMP-2 treatment. The altered genes included Wnt pathway receptors, as well as positive and negative regulators of the Wnt signaling pathway (Fig. 7B). Real-time PCR analyses further confirmed the altered expression of several key Wnt pathway associated genes in *Cox-2^f/f^; Prx1Cre* PDMPCs, namely, *Wif-1*, *N-cadherin*, *LRP4*, *FRZB*, *and TCF7* (Fig. 7C).

## Discussion

During fracture healing mesenchymal progenitors residing in periosteum undergo osteogenic and chondrogenic differentiation to induce intramembranous and endochondral bone formation. To understand the spatiotemporal control of periosteal mesenchymal progenitor cell differentiation during repair and regeneration, we specifically deleted the *Cox2* gene in mesenchyme via Prx1cre or in cartilage via Col2Cre. Our studies show that Cox-2 acts at the early mesenchyme differentiation stage and mediates both osteogenic and chondrogenic differentiation of PDMPC during repair. Targeted *Cox-2* deletion via Prx1Cre in mesenchyme disrupted the entire differentiation program of mesenchyme progenitors, leading to reduction of bone formation and accretion of mesenchyme and immature cartilage in the callus (Figure 1). By contrast, targeted *Cox-2* deletion via *Col2Cre* expression in cartilage impaired fracture healing primarily by disrupting chondrocyte maturation, vascular invasion and endochondral bone formation. Consistent with the *in vivo* observation, cell differentiation and gene profiling analyses showed that *Prx1Cre*-mediated *Cox-2* deletion blunted osteogenic PDMPC differentiation, attenuated chondrogenesis and blocked BMP-2-induced chondrocyte maturation and terminal differentiation. Our data are consistent with the previous observation which demonstrates a marked induction of *Cox-2* mRNA at the onset of endochondral and intramembranous repair in early fracture callus [14], [15], further providing direct evidence for a unique spatiotemporal role of Cox-2 in osteogenic and chondrogenic differentiation of periosteal progenitor cells in bone repair and regeneration.

To obtain mechanistic information underlying impaired fracture healing in Cox-2 deficient mice, we utilized a previously established method which allows isolation and *in vitro* analyses of mesenchymal progenitors derived from autografted periosteal callus (PDMPCs) [17], [18], [24]. This procedure permits robust isolation and recovery of otherwise limiting cell populations for biochemical and molecular analyses. By analyzing the differentiation potential and gene profiling of PDMPCs obtained directly from the healing site, we demonstrated that Cox-2 deficient cells exhibited decreased osteogenic and chondrogenic differentiation potential under basal culture conditions, for both monolayer and micromass culture. Gene profiling analyses revealed significant down-regulation in Cox-2 deficient cells of a set of key genes that control bone/cartilage ossification and remodeling as compared to the wild type controls; the genes *Sox9*, *Sp7 (OSX)*, *MMP13*, *MMP9*, *RANKL* and *VDR*. The reduced expression of this key set of genes in Cox-2 deficient mesenchymal progenitors is likely to explain the impaired bone formation and delayed cartilage remodeling observed in the Cox-2 mutant mice at the onset of fracture healing, indicating that the differentiation of the mesenchymal progenitors depends on Cox-2 expression during initiation of healing. The data are consistent with the anabolic effects of prostaglandins, e.g. prostaglandin E2 (PGE2) in stimulating bone formation and bone/cartilage remodeling in repair [15], [25]–[27], underscoring a direct role of Cox-2 from cells of mesenchymal lineages in modulating expression of this key set of genes in repair and regeneration. In addition to the altered gene expression associated with bone formation, GO analyses also identified functional gene clusters (Table S1) that regulate immune and inflammatory responses, suggesting that Cox-2 deletion in mesenchymal progenitors could further modify immune response and change local inflammatory microenvironments at the onset of bone healing [28], [29].

By analyzing PDMPC differentiation in response to BMP-2 treatment, our study demonstrated a key role of Cox-2 in BMP-2-induced mesenchymal differentiation. BMP-2 is known for its strong osteo-inductive and chondro-inductive actions on mesenchymal progenitors *in vivo* and *in vitro* [30]. Recent studies have further established BMP-2 as a critical gene in the initiation of bone fracture repair [31]. Similar to Cox-2, BMP-2 deletion via Prx1Cre produces minimal effects on embryonic long bone development (23). However, postnatal deletion of the BMP-2 gene in periosteum impairs chondrogenic and osteogenic differentiation of mesenchymal progenitor cells and impedes periosteum-mediated endochondral and intramembranous bone formation [17], [32]. While we observed modest Cox-2 protein induction in monolayer cultures, both Cox-2 protein and mRNA were markedly induced by BMP-2 in micromass cultures, suggesting a key role of Cox-2 in BMP-2 mediated chondrogenic differentiation of PDMPCs. Prx1Cre-mediated *Cox-2* deletion further attenuated BMP-2-induced osteogenic differentiation in monolayer culture and completely blocked chondrocyte maturation and terminal differentiation in micromass culture. These data speak directly to the mechanism by which BMP-2 mediates bone differentiation, and establishes Cox-2 as a critical downstream mediator of BMP-2 action, demonstrating an important role of the BMP-2/Cox-2 axis in control of chondrogenic and osteogenic differentiation of mesenchymal progenitors in postnatal bone tissue repair.

By using GO pathway enrichment analyses, we identified the phosphoinositide 3-kinase/protein kinase B (PI3K/AKT), Hypoxia Inducible Factor-1 (HIF-1) and the Wnt pathway as key signaling pathways targeted by Cox-2 in BMP-2-induced PDMPC differentiation. The PI3K/AKT pathway crosstalks with a number of signaling pathways, including BMP/TGFβ signaling pathway, mTOR, NF-κb, JAK/STAT, MAPK, CREB, P53 and VEGF, which are known for their roles in stem/progenitor cell proliferation, osteoblast and chondrocyte differentiation, apoptosis and angiogenesis [33]–[35]. The PI3K/AKT pathway also plays a role in regulating glycolysis and gluconeogenesis processes [36], [37], which are markedly affected by Cox-2 deletion (Table 1). A link between Cox-2 and the PI3K/AKT pathway has recently been reported in mouse and human osteoblasts [38]. Downregulation of COX-2 via gene silencing suppresses phosphorylation of AKT and PTEN. Interestingly, PGE2, one of the potential downstream products resulting from cyclooxygenase activity, failed to reverse COX-2-dependent AKT phosphorylation, suggesting a potential PGE2 independent mechanism(s) in BMP-2/COX-2/PI3K/AKT-mediated regulation of cell differentiation.

The HIF-1 pathway plays a central role in cellular response to hypoxic condition and is essential for bone/cartilage development and chondrocyte survival [39]. The HIF-1 pathway is also critically important in bone repair and regeneration [40], [41]. While a direct link between HIF-1 pathway and Cox-2-mediated repair remains to be established, hypoxia regulates PGE2 release in osteoblasts [42], [43] and COX-2/PGE2 signalling is involved in a hypoxia-induced angiogenic response in endothelial cells [44].The central player of HIF-1 pathway is HIF-1α, which is regulated at the post-transcriptional level by the HIF prolyl-hydroxylase domain enzymes (PHDs) (gene name: Egl nine homologs, Eglns). Eglns hydroxylate the α-subunit of HIF-1α, enabling binding of the von Hippel-Lindau (VHL) protein for poly-ubiquitination, which ultimately leads to proteolytic proteasomal degradation of HIF-1α [45], [46]. In our current study, HIF-1α expression was only modest regulated during chondrogenic differentiation. However, the HIF prolyl-hydroxylase domain enzymes Egln1 and Egln3 were markedly induced by BMP-2 in PDMPCs at day 7, and this induction was abolished in the *Cox-2* deficient cells. The data suggest a requirement for Cox-2 expression in BMP-2 induction of *Egln1* and *3*, and their likely subsequent involvement in chondrocyte differentiation, vascular invasion and endochondral bone formation. In addition to *Egln1* and *Egln3*, a subset of genes associated with hypoxia, angiogenesis and vasculogenesis, namely *VEGFA* and *Angiopoietin 4* (*ANGPT4*), *Ddit4*, *Eif4ebp1*, *Camk2b*, *Pfkl*, *Ldha*, *Aldoa*, *PDK1*, *Slc2a1* were also markedly suppressed in *Cox-2* deficient PDMPCs (Fig. 6B). These data suggest a central role for Cox-2 from mesenchymal lineage in coordinating osteogenesis and angiogenesis in response to hypoxia during endochondral bone repair.

The Wnt pathway is known to play key roles in bone and cartilage development [47]. Canonical Wnt pathway activation favors osteoblastic differentiation, but inhibits chondrogenesis [48], [49]. Activation of β-catenin signaling further stimulates chondrocyte hypertrophy and vascular invasion [50]. Although detailed molecular actions of the Wnt pathway on different phases of endochondral bone repair remain to be determined, genetic manipulation of Wnt signaling in mice demonstrates that inhibition of Wnt/β-catenin expression suppresses early chondrogenesis but favors osteogenesis, leading to accelerated but reduced fracture repair [51]–[53]. In our current study, we noted that Cox-2 inactivation in PDMPCs down regulated a group of genes that are classified as negative regulators of the canonical Wnt pathway; e.g., *N-cadherin*, *FRZB* and *Sfrps*, along with Sox9 at day 1 (Fig. 7A) Contrary to accelerated repair observed in a Wnt/β-catenin gain-of-function mouse model, Cox-2 deficiency is associated with a marked reduction of osteogenesis *in vitro* and *in vivo*, indicating that Cox-2 orchestrates a complex signaling interplay in conjunction with Wnt pathway regulators during repair and regeneration.

NSAIDs (non-steroidal anti-inflammatory drugs) which often inhibit both Cox-1 and Cox-2 are well known as having a negative effect on fracture healing in rat models [54], [55]. Prolonged use of targeted Cox-2 inhibitors delays fracture healing in rats [13]. Transient inhibition of Cox-2 has small and reversible effects on fracture healing, suggesting that the adverse effect of Cox-2 inhibition may be both dosage and duration dependent [56], [57]. Pharmacological inhibition of Cox-2 activity is also reported to have an inhibitory effect on differentiation of human and mouse mesenchymal stem cells [58], [59]. Pharmacological studies are often confounded by dosing issue, specificity and the potential off-targeting effects of the drug. Using a gene targeting approach, our current study moves the field forward, we believe, by identifying cells that are likely to be the specific (or a specific) cell type in which COX-2 expression plays a modulatory role in fracture repair, and demonstrates the consequences of targeted Cox-2 gene deletion on fracture repair *in vivo*.

In summary, using cell-type specific *Cox-2* gene deletion, we demonstrate a spatial and temporal role for Cox-2 function in endochondral and intramembranous bone repair; targeted *Cox-2* gene deletion inhibits BMP-2-induced osteogenic, chondrogenic and angiogenic responses in periosteum-derived mesenchymal progenitors. Gene profiling analyses uncovered Cox-2-targeted pathways, e.g., the HIF-1, PI3K/AKT and Wnt pathways that modulate periosteal progenitor cell differentiation in bone fracture repair and regeneration. Identification of critical genes/targets in periosteal-derived mesenchymal progenitor cell differentiation could assist in identification of novel drug targets, facilitating development of new therapeutic solutions for bone repair and regeneration.

## Supporting Information

Figure S1 **Cox-2^f/f^; Prx1cre mice have normal long bone length and cortical bone morphology.** Quantitative measurements show identical length (A) and cortical thickness (B) in tibias of two-month-old Cox-2^f/f^ and Cox-2^f/f^; Prx1Cre mice. Data are presented as means ± SEM, n = 6.
(TIF)

Figure S2 *Cox-2* mRNA expression in day 1 and day 7 micromass cultures. Real Time PCR analyses show robust induction of Cox-2 gene expression by BMP-2 in micromass culture. Cox-2 mRNA was reduced by more than 95% in the Cox-2^f/f^; Prx1Cre cells isolated from periosteal callus. * p<0.05, as compared to the control.
(TIF)

Figure S3 Comparison of Cox-2^f/f^ (WT) and Cox-2^f/f^; Prx1cre PDMPC gene expression profiles at day 1 vs. day 7 identified 1159 differentially expressed genes that exhibited a change of 2 fold or more. Hierarchical clustering analyses were used to generate the heat maps showing expression of these genes in Cox-2^f/f^ (WT) and Cox-2^f/f^; prx1cre (KO) cells at day 1 and 7 (A). Subsets of genes (111genes) associated with bone/cartilage formation and mineralization in Cox-2^f/f^ and Cox-2^f/f^; prx1cre cells at day 1 and 7 are further illustrated in the heat maps generated by hierarchical clustering analyses (B). The suppressed genes in Cox-2^f/f^; prx1cre (KO) cells as compared to the Cox-2^f/f^ (WT) cells at day 7 are listed in no particular order at the bottom. Gene up-regulation is presented in red and gene down-regulation is in blue.
(TIF)

Figure S4 Heat map showing expression of 1183 BMP-2 responsive genes in Cox-2^f/f^ (WT) and Cox-2^f/f^; prx1cre cells (KO) at day 7 (A). Among them, 181 unique probes associated with bone/cartilage formation and mineralization in Cox-2^f/f^ (WT) and Cox-2^f/f^; prx1cre (KO) cells were subjected to hierarchical clustering analyses to generate a heat map (B). Thirty-nine genes representing significantly suppressed genes in the KO cells at basal level or following BMP-2 treatment are listed at bottom without particular order. Gene up-regulation is presented in red and gene down-regulation is in blue.
(TIF)

Table S1 **Go classification of the differentially expressed genes in Cox-2 deficient cells at day 1 without any treatment.** Table S1 lists major functional categories enriched by DAVID using differentially expressed genes in Cox-2 deficient cells at day 1 without BMP-2 treatment. Genes suppressed or increased by 2 fold or more in the absence of Cox-2 were separately analyzed. Fisher exact P values for the gene-enrichment categories were generated from a reference gene list provided by Partek Genomic Suite software. “Genes included in the group” indicate the number of genes enriched for that category from the input gene list.
(DOCX)

Table S2 **Additional RT-PCR primers used for RT-PCR analyses in this study are listed.**
(DOCX)
