# Supplementary figures and images for: The Spatiotemporal Role of COX-2 in Osteogenic and Chondrogenic Differentiation of Periosteum-Derived Mesenchymal Progenitors in Fracture Repair
Source: PLoS One. 2014 Jul 2;9(7):e100079. doi: 10.1371/journal.pone.0100079 (PMC4079554; doi:10.1371/journal.pone.0100079)

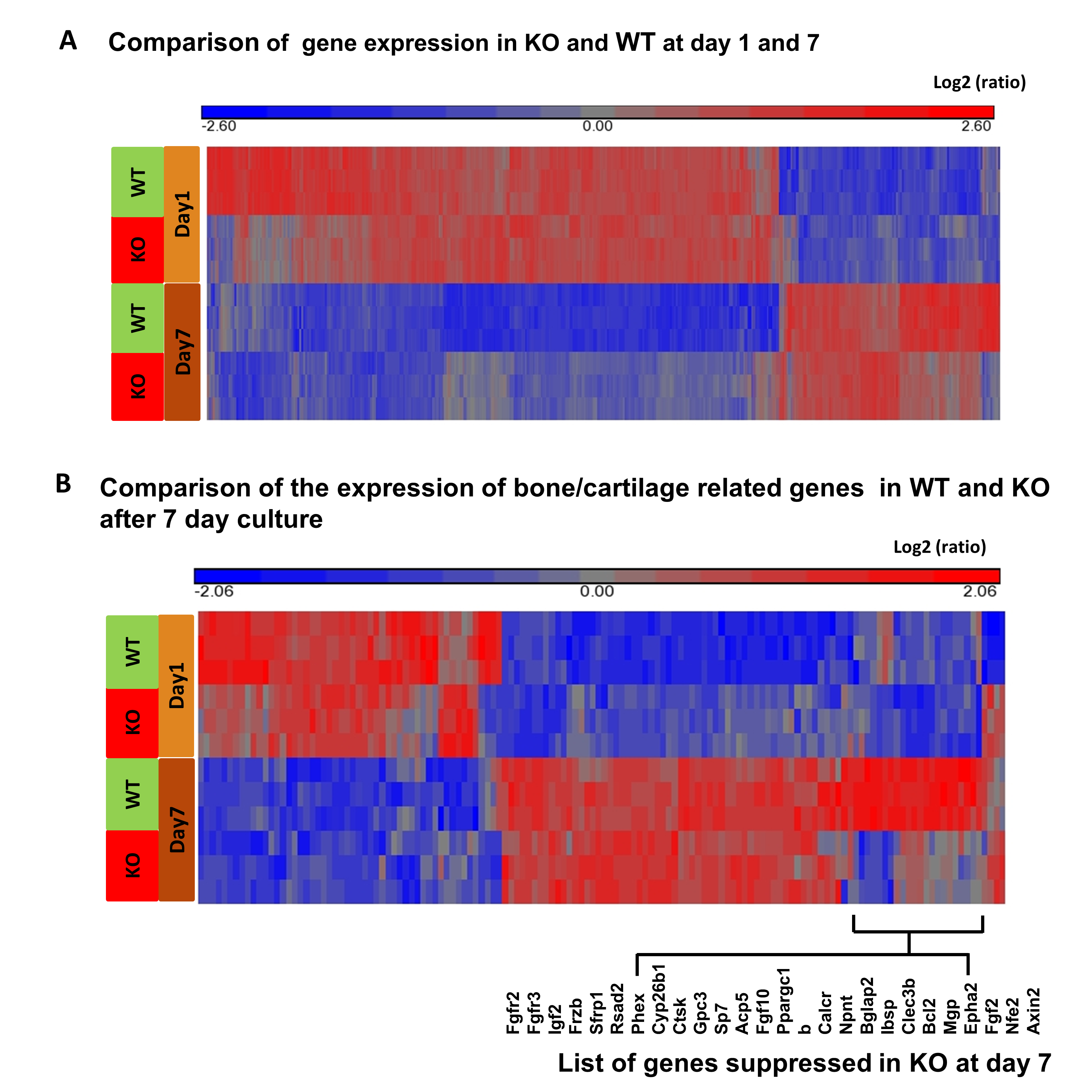

Supplement: Figure S3 — Comparison of Cox-2f/f (WT) and Cox-2f/f; Prx1cre PDMPC gene expression profiles at day 1 vs. day 7 identified 1159 differentially expressed genes that exhibited a change of 2 fold or more. Hierarchical clustering analyses were used to generate the heat maps showing expression of these genes in Cox-2f/f (WT) and Cox-2f/f; prx1cre (KO) cells at day 1 and 7 (A). Subsets of genes (111genes) associated with bone/cartilage formation and mineralization in Cox-2f/f and Cox-2f/f; prx1cre cells at day 1 and 7 are further illustrated in the heat maps generated by hierarchical clustering analyses (B). The suppressed genes in Cox-2f/f; prx1cre (KO) cells as compared to the Cox-2f/f (WT) cells at day 7 are listed in no particular order at the bottom. Gene up-regulation is presented in red and gene down-regulation is in blue. (TIF) [file pone.0100079.s003.tif]

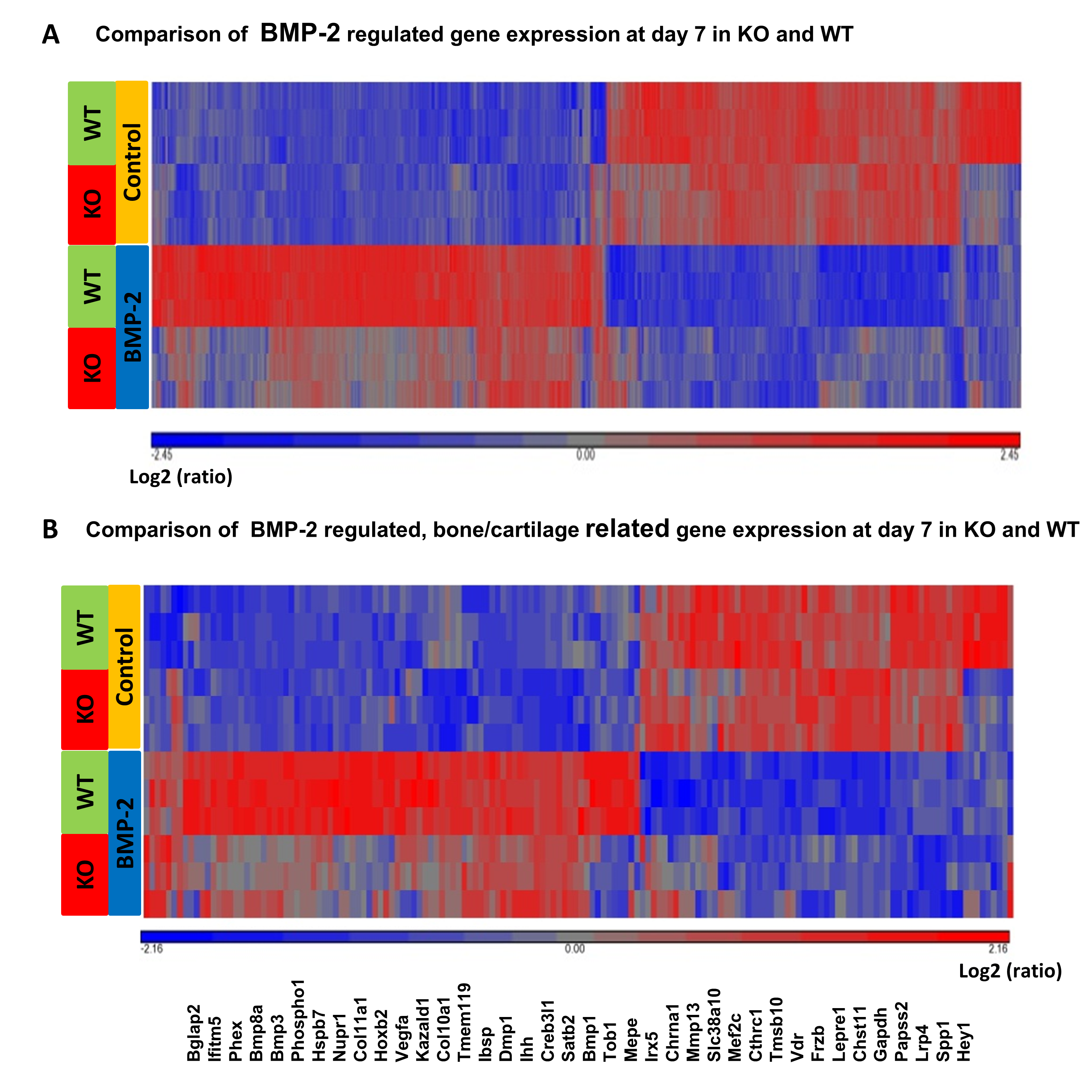

Supplement: Figure S4 — Heat map showing expression of 1183 BMP-2 responsive genes in Cox-2f/f (WT) and Cox-2f/f; prx1cre cells (KO) at day 7 (A). Among them, 181 unique probes associated with bone/cartilage formation and mineralization in Cox-2f/f (WT) and Cox-2f/f; prx1cre (KO) cells were subjected to hierarchical clustering analyses to generate a heat map (B). Thirty-nine genes representing significantly suppressed genes in the KO cells at basal level or following BMP-2 treatment are listed at bottom without particular order. Gene up-regulation is presented in red and gene down-regulation is in blue. (TIF) [file pone.0100079.s004.tif]
