# Supplementary material for: The Spatiotemporal Role of COX-2 in Osteogenic and Chondrogenic Differentiation of Periosteum-Derived Mesenchymal Progenitors in Fracture Repair
Source: PLoS One. 2014 Jul 2;9(7):e100079. doi: 10.1371/journal.pone.0100079 (PMC4079554; doi:10.1371/journal.pone.0100079)
Supplement: Table S1 — Go classification of the differentially expressed genes in Cox-2 deficient cells at day 1 without any treatment. Table S1 lists major functional categories enriched by DAVID using differentially expressed genes in Cox-2 deficient cells at day 1 without BMP-2 treatment. Genes suppressed or increased by 2 fold or more in the absence of Cox-2 were separately analyzed. Fisher exact P values for the gene-enrichment categories were generated from a reference gene list provided by Partek Genomic Suite software. “Genes included in the group” indicate the number of genes enriched for that category from the input gene list. (DOCX) [file pone.0100079.s005.docx]

| **Functional classification**  **Table S1. Go classification of the differentially expressed genes in Cox-2 deficient cells at day 1 without treatment** | **Fisher exact**  **p value** | **Genes included in the group** |
| --- | --- | --- |
| **Genes suppressed by 2-fold or more in KO compared to the WT (143 genes)** | | |
| Bone development/ossification process (ossification) | 4.0E-5 | 11 |
| Immune response/inflammatory response | 8.2E-5 | 21 |
| Growth factor activity | 3.3E-4 | 7 |
| Wnt pathway | <0.001 | 6 |
| Morphogenesis of a branching structure | <0.005 | 7 |
| **Genes increased by 2-fold or more in KO compared to the WT (242 genes)** | | |
| Immune/inflammatory response | 9.6E-4 | 60 |
| Blood vessel development | <0.01 | 16 |
| Cell migration and chemotaxis | <0.01 | 19 |
