# Supplementary material for: The Spatiotemporal Role of COX-2 in Osteogenic and Chondrogenic Differentiation of Periosteum-Derived Mesenchymal Progenitors in Fracture Repair
Source: PLoS One. 2014 Jul 2;9(7):e100079. doi: 10.1371/journal.pone.0100079 (PMC4079554; doi:10.1371/journal.pone.0100079)
Supplement: Table S2 — Additional RT-PCR primers used for RT-PCR analyses in this study are listed. (DOCX) [file pone.0100079.s006.docx]

| **Name** | **Sequence** |
| --- | --- |
| EGLN3-F, | AAGTTAGCCACCCTCGTTTC |
| EGLN3-R | CTATGCTGCTTGTGGGATTCTA |
| EGLN1-F | CGTCTTAGCACCTGTGTAGTTT |
| EGLN1-R | CACGGCTCTACTTGGTTGTT |
| HIF1a-F | TCACAGCTCCTCAGCATTT |
| HIF1a-R | GGACAAACTCCCTCACCAAA |
| CDH2-F | GGATGAAACGGCGGGATAAA |
| CDH2-R | TCTTCTTCTCCTCCACCTTCTT |
| LRP4-F | CTCTTCCATTCTGTGGGATTCT |
| LRP4-R | TGTGTCTCTCTAGTTGCTCTTTC |
| TCF 7-F | CCAAGAAGCTCACCAGCATTA |
| TCF 7-R | TGTGTCTCTCTAGTTGCTCTTTC |
| FRZB-F | CGTTCCAGGTTACTCTTGGTAG |
| FRZB-R | CCAAGGTGTCGGAGTTTCATA |
| COX-2-F | ACAGTCCACCTACTTACAATGTG |
| COX-2-R | TCATCTGCTACGGGAGGAA |

**Table S2. Additional RT-PCR primers used in this study**
